# The effect of mobile health technology on blood pressure control among patients with hypertension in Ghana and Nigeria

**DOI:** 10.21203/rs.3.rs-3272069/v1

**Published:** 2023-09-11

**Authors:** Bolade Folasade Dele-Ojo, Tijani Idris Ahmad Oseni, Fiifi Duodu, Chidiebere Peter Echieh, Paa-Kwesi Blankson, Biodun Sulyman Alabi, Daniel F Sarpong, Bamidele O Tayo, Vincent Boima, Mary Amoakoh Coleman, Gbenga Ogedegbe

**Affiliations:** Ekiti State University; Ambrose Alli University; Korle Bu Teaching Hospital; University of Arizona Medical Center; Korle Bu Teaching Hospital; University of Ilorin; Yale New Haven Hospital; Loyola University Medical Center; University of Ghana; Noguchi Memorial Institute for Medical Research; New York University

**Keywords:** Hypertension, mobile health intervention, systolic blood pressure reduction, blood pressure control, adherence

## Abstract

**Background:**

More than half of patients with hypertension in sub-Saharan African do not achieve blood pressure control. This study determined the effect of mobile health technology on systolic blood pressure reduction and blood pressure (BP) control among patients with hypertension in Nigeria and Ghana.

**Methods:**

A randomised control trial of 225 adults with hypertension attending two General/Medical Outpatient Clinics each in Nigeria and Ghana was randomized into intervention (n = 116) and control (n = 109) arm respectively. Patients in the intervention arm received messages twice weekly from a mobile app for six months in addition to the usual care while the control arm received usual care only. The study outcomes were systolic blood pressure (SBP) reduction and blood pressure control at six months, while the secondary outcome was medication adherence at six months. Data were collected at 0 and 6 months, it was analysed using SPSS-21 software at a significance level of p < 0.05. Binary logistic regression was used to generate the predictors of good blood pressure control.

**Results:**

The mean age for the control and intervention were 60.2 ± 13.5 and 62.6 ± 10.8 years respectively; p-value = 0.300. The intervention group had greater reductions in SBP (−18.7mmHg vs −3.9mmHg; p < 0.001) and greater BP control rate (44.3% vs 24.8%; p-value 0.002).

**Conclusions:**

The mobile health intervention resulted in significant SBP reduction rate and improvement in BP control rate in the 6th month. However, improvement in adherence level in the 3rd month and was not sustained in the 6th month. The addition of mobile health technology may be extended for use in the national hypertension control plan. Female gender, formal education and being in the intervention arm were predictors of blood pressure control.

## Background

Hypertension is the leading risk factor for cardiovascular disease and a major contributor to disease burden and disability worldwide^1,2^. Individuals with uncontrolled hypertension who do not comply with recommended treatment strategies are at increased risk of strokes, myocardial infarction and chronic kidney disease, which may lead to hospitalization and consequently greater healthcare costs^3,4^. Unfortunately, high rates of uncontrolled blood pressure (BP) have been reported among patients with hypertension accessing healthcare in both countries, with 56% of patients with hypertension in sub-Saharan Africa (SSA) not achieving control despite accessing healthcare facilities and the availability of multiple treatment options^5,6^. Some of the contributory factors to poor BP control are non-adherence to regular antihypertensive medications, clinic appointments, healthy diet and lifestyle modifications. Hence, engaging more active patients and physicians’ involvement in hypertension management through mobile health technologies promises to improve BP control^7^.

Coincidentally, there has been a remarkably rapid increase in smartphone devices globally, over the past decades, with SSA not exempt from this revolution. It has been estimated that by 2022, there will be 6.8 billion smartphone users, globally^8^. The penetration of mobile phones has corresponded with an increase in health-related applications which offer health services and information, referred to as mobile health (mHealth)^9^. Mobile phone technology, therefore, seems to provide a potential medium to support direct healthcare delivery and patient-provider communication. Though, mobile-health technologies have been used with success in other medical areas^10^. In Ghana, Sarfo *et. al* demonstrated an improvement in BP control among stroke survivors in a resource-limited setting via a mHealth intervention^11^. In Nigeria, previous studies in other fields of Medicine aside from cardiology have shown desirable effects in the intervention arms^8–10^. Otieno *et. al* launched mHealth in rural and urban areas of Ghana and Kenya and subsequently demonstrated an increment in the proportion of patients with controlled blood pressure from 46% at baseline to 77% at 12 months^12^. Generally, there is limited research on the use of mHealth for disease diagnosis and treatment support in sub-Saharan Africa^13^. Therefore, this study determined the effect of mobile health interventions on systolic blood pressure reduction, the rate of blood pressure control and adherence to scheduled clinic appointments, diet and medications among patients with hypertension in Nigeria and Ghana.

## Methods

### Study design, setting and participants

The study was a randomized clinical trial involving four study sites with similar institutional and population characteristics in Nigeria and Ghana. The sites were selected through purposive sampling and were the Outpatient Department (OPD) clinics of Mamprobi Hospital (MH) and the Adabraka Polyclinic (AP) in Ghana and the Outpatient Department (OPD) clinics of Ekiti State University Teaching Hospital (EKSUTH), Ado-Ekiti and the Federal Teaching Hospital (FTH), Ido-Ekiti in Nigeria. A total of 225 adults with essential hypertension, receiving antihypertensive medications, who owned a smartphone, with at least basic formal education and the ability to read and understand English were systematically selected from the four study sites and 116 of them were randomized into intervention arm and the 109 into control arm. However, patients with self-reported secondary hypertension; self-reported renal disease; physical or mental disability that impaired use of the mobile application; and severely ill or weak patients whose condition would not allow them to be interviewed at review dates were excluded from the study.

#### Sample size determination:

The sample size was determined at a confidence level of 95% using the single sample for infinite population formula: Ss = (z2 × σ2)/d2^14^, where Z = Z value (e.g. 1.96 for 95% confidence level); σ = Standard Deviation; d = distance on either side of mean in confidence interval. Sarfo et al. in a study to ascertain the effect of BP reduction and medication adherence using mobile-health (mHealth) technology among Ghanaian stroke survivors found the mean adherence ratio to be 0.95 ± 0.16 15^11^. Using the standard deviation from Sarfo et al., and assuming d of 0.05, the sample size was calculated as [1.962 × 0.162] / 0.052 = 39.34 which approximates to 39. Assuming an attrition rate of 20%, (which approximates to 8), the minimum sample size was calculated to give 47 from each study site with the intervention and control arm having 94 participants each making a total of 188. However, 116 participants in the intervention arm and 109 in the control arm completed the study respectively giving a total of 225 participants. This was done to increase the power of the study.

#### Randomization:

Patients who met the selection criteria and consented to participate in the study were randomised into Intervention and Control Groups in a 1:1 ratio using a computer-generated sequence placed in opaque envelopes numbered serially and with cards to indicate whether a patient should be in the intervention or control arm after matching for sociodemographic characteristics like age, sex, education, income level and BMI. They were then used to allocate patients by an independent research assistant. Data collection was done at the clinics before patients consulted with their Physicians. Structured questionnaires were used for data collection and included background characteristics (age, sex, marital status, employment status, income, occupation and health insurance scheme enrolment), BP measurements, medication adherence and lifestyle.

### Data Collection

All participants (intervention and control arms) received standard routine care for the management of hypertension in the clinics which included history, examination, investigations, medications, and health education at the various clinics on healthy lifestyle choices and regular clinic visits. However, the participants in the intervention group received mHealth in addition to the standard care, while those in the control group received the standard care alone.

### Anthropometric and blood pressure measurements

Clinical measurements of weight, height, waist and hip circumferences and blood pressure were obtained by standard protocols. Weight to the nearest 0.1kg while the participants were barefooted, and height to the nearest 0.1m without head-gear, were determined with bathroom scales and stadiometer respectively. The BMI (Body Mass Index) was taken as the ratio of weight to the square of the height, while the Waist-to-Hip Ratio (WHR) was calculated from the values of waist and hip circumferences. BP readings were taken three (3) times at an interval of a minimum of 10 minutes using OMRON M3 digital sphygmomanometers. The mean systolic and diastolic values were determined using the last two BP readings. Based on the Joint National Committee on Prevention, Detection, Evaluation, and Treatment of High Blood Pressure- Seventh Report (JNC-7) guidelines^15–17^. This study considered patients with BP of < 140/90mmHg as controlled while those with BP ≥ 140/90mmHg were categorized as uncontrolled.

### Mobile Health Interventions

The mHealth intervention consisted of an interactive phone application (m-notify^®^) that sent reminders to the participants in the intervention group on antihypertensive medication usage, clinic appointments and healthy lifestyle choices such as low salt diet, smoking cessation, alcohol reduction and need for regular physical activity for at least 30 minutes daily for 5 or more times per week. These messages were sent twice weekly throughout the duration of the study (6 months). An example of a message sent was

Walk briskly for at least 30 minutes a day for a minimum of 5 days a week for a healthy heart,

Salt is hidden in a lot of processed food, check food labels and choose food with little or no salt. Also, don’t add salt to food after serving as salt worsens hypertension.

The m-notify App gives feedback that respondents got the messages and read them.

### Hill-Bone adherence scale

This was used to assess adherence to scheduled clinic appointments, diet and medications. Hill-Bone adherence scale is a 14-item scale; appointment-keeping (2 items), diet (3 items) and medication adherence (9 items)^18^. Each item was scored based on the response on a scale of 1 to 4. A score of 4 indicates the poorest compliance while 1 indicates the best compliance. After adding the points of all 14 items, the total score ranged from 14 to 56. The participant’s total score was converted to percentages and those with < 80% were categorised as non-adherent while those with 80% and above were categorised as being adherent.

The study was carried out from May 2021 to December 2021.

### Follow-up

The study outcomes were re-assessed at the 3rd and 6th month of follow-up for all the participants in the intervention and control arms.

### Statistical analysis

Data were analysed using Statistical Package for Social Sciences (IBM SPSS) version 25 for Windows (IBM Corp., Armonk, N.Y., USA). Data were collected at baseline, 3rd month and the 6th month. Continuous variables were expressed as Means Standard Deviation (SD) and compared with Students t-test while categorical variables were expressed as frequency (percentages) and Pearson’s Chi-square was used to compare the differences in the frequency distribution of the categorical variables. Binary logistic regression was used to generate the predictors of good blood pressure control and the level of significance was set at two-sided p-value < 0.05.

## Results

The sociodemographic characteristics of the study population were shown in Table 1. A total of 225 subjects were recruited for the study across the two countries, which consisted of 109 participants in the control and 116 participants in the intervention arm. The mean age for the control and intervention were 60.2 ± 13.5 and 62.6 ± 10.8years respectively. Males constituted 37.6% and 35.3% in the control and intervention arms respectively. Health insurance coverage was similar in both groups. However, monthly income was significantly higher in the intervention arm than in the control arm. Also, 63 (54.3%) of the participants in the intervention arm had tertiary education compared with 21 (19.3%) of the control arm. In addition, 32 (27.6%) of the participants in the intervention arm were government employees compared with 8 (7.3%) in the control arm.

### Rates of SBP reduction in 6th month

In the control arm, the mean SBP reduction rate (SD) in the control group was 2.2 (7.8%) while in the intervention arm, the mean SBP reduction rate (SD) was 11.2 (10.2%). Table 2 shows the comparison of the clinical parameters between control and intervention, pre-intervention and post-intervention. There was no statistically significant difference between the two groups in the pre-intervention phase, but after the intervention, systolic and diastolic BP levels were significantly lower in the intervention group than in the control group (p < .0.001/ 0.032) in the 6th month.

Table 3 shows the comparison of BP control between the intervention and control groups at baseline, 3rd month and 6th month. There was no statistically significant difference between the control and intervention groups in the pre-intervention phase 17.4% vs 11.2%; p-value = 0.182. However, in the 6th month, a greater proportion of the intervention group had good BP control compared with the usual care: 44.3% versus 24.8%; p-value = 0.002.

Table 4 depicts the comparison of the Medication Adherence Level of the Respondents in the Intervention and Control groups at the Baseline, 3rd month and 6th month in Ghana and Nigeria. The pooled data, at baseline,58 (53.2%) of the control and 58 (50.0%) of the intervention group had good adherence respectively. The difference was not statistically significant (p = 0.566) However, at the 3rd month, a greater percentage of the intervention group 78 (67.3%) had good adherence than 63 (57.8%) of the control group; p-value = 0.029. Meanwhile, in the 6th month, the significant difference as regards good adherence between the 2 groups was blunted.

Table 5 shows the relationship between good blood pressure control and the socio-demographic variables. At the end of the 6 months, there was a significant improvement in blood pressure control among participants who were females (p = 0.017), with tertiary level of education (p = 0.010) and in the intervention arm of the study (p = 0.002).

Table 6 shows the predictors of BP control. Female gender, formal education and being in the intervention arm were predictors of blood pressure control: Females were 2.5 times more likely to have good BP control; AOR = 2.52, CI = 1.33–4.79, Participants with secondary and tertiary education were about 17 times (AOR = 16.61, CI = 2.06–133.91 ) and 15 times (AOR = 15.07, CI = 1.86– 121.76) more likely to have good blood pressure respectively compared with those without formal education; while those in the Intervention arm were twice more likely to have good blood pressure (AOR = 2.33, CI = 1.23–4.42).

## Discussion

Mobile health technologies improve hypertension outcomes^7^ and they can be effective in self-management of chronic diseases^13,19^. Therefore, this study showed that the addition of mHealth to usual care among patients with hypertension led to significant reductions in SBP and DBP. Our findings are in tandem with previous studies that revealed that the mHealth intervention led to greater reductions in systolic and diastolic BP than usual care ^15–17,20^. Additionally, this study showed that the addition of mHealth to the usual care among patients with hypertension led to an increase in the proportion of those with good BP control in the intervention arm versus the control group (44.3% vs 24.8%, p-value = 0.002) in the 6th month. Similarly, a meta-analysis and systematic review carried out among adult Chinese revealed that mHealth interventions led to an improvement in BP control^21^. Our findings are also in tandem with a similar study by Otieno *et. al.,* in the rural and urban areas of Ghana and Kenya which demonstrated an increment in the proportion of patients with controlled pressure from 46% at baseline to 77% at 12 months^12^. In our study, the proportion of increase in both studies was similar (over 30% with the use of mHealth). Nonetheless, the proportion of good BP control in our study was very low at baseline (11.2%). Optimal control of BP is still poor in our settings; Abdu *et al* in Nigeria reported 27.6% of their study population had good BP control^22^ while Boima *et al.* in Ghana reported poor BP control in 69.7% of their participants^23^. The high rate of uncontrolled hypertension has been attributed to poor medication adherence and inadequate health insurance coverage ^23^.

Furthermore, adherence to scheduled clinic appointments, healthy diet and medications were assessed using the Hill-Bone Adherence Scale in this study. A high proportion of non-adherence in both intervention and control groups at 46.8% and 50% respectively were reported at baseline. This is similar to previous studies which also reported a high proportion of medication non-adherence among hypertensives in Ghana and Nigeria^23, 24^. However, the addition of mHealth showed an initial beneficial effect in 3rd month, as the proportion of good adherence in the intervention arm increased significantly from 50% at baseline to 67.3% while the control group barely increased from 53.2% at baseline to 57.8% at the 3rd month. A similar study has shown a rise in adherence with the use of mHealth^1,15^. However, the significant improvement in the adherence level of the intervention group in comparison with the control group in the 3rd month, but was blunted in the 6th month. The reasons for non-adherence to treatment in our settings could be due to financial constraints, lack of health insurance, side effects of the medications and then religious and socio-cultural factors^25^. Besides, the success of hypertension therapy is not only dependent on the healthcare systems and healthcare professionals alone but also on the support of friends/family especially to maintain long-term medication adherence, maintaining scheduled follow-ups level and healthy diets^26^. Another study revealed the involvement of family members was a determinant of good hypertension self-care^27^. The most important outcomes of the study include adherence to treatment, weight control, and regular monitoring of blood pressure which are assessed in the primary assessment. The intervention is a mobile application that has capabilities such as reminders and scientific and supportive information. This application has been programmed to reduce many of the non-adherence factors of hypertension treatment. Therefore, the findings may contribute to a rise in adherence to treatment. If proven to have an appropriate impact, it may be extended for use in the national hypertension control plan. Therefore, this study has revealed a gap in the need to involve family members and friends in the management of patients with hypertension, if there must be a long-term improvement in adherence to healthy diets, medication and clinic appointments. There was a decline in adherence in both groups at the 6th month, this could likely be due to inertia to the use of mHealth. Besides, behaviour change is key to the long-term adoption of a healthy and active lifestyle^28^. For lifestyle interventions such as structured exercise interventions to be adopted by patients, practitioners need to ensure that behaviour change programmes are mapped against patient’s priorities and values, and adapted to their level of readiness and intention to engage with the target behaviour.

Furthermore, at the end of our study, in 6th month, female gender, formal education and being in the intervention arm were predictors of good blood pressure control: Females had 2.5 times likelihood of having good BP control; having formal education; Primary school education had about 9 times, secondary school education had about 17 times and tertiary education had 15 times likelihood of having good blood pressure than those without formal education while those in the Intervention arm were twice more likely to have good blood pressure than those in the control arm. Females have been shown to have lower BP than Males^29^. Furthermore, a previous study has also shown that the university educational level of the participants as well as receiving health education, by healthcare providers and family members were predictors of good hypertension self-care, which will also result in good BP control^27^. In our study, having health insurance coverage and monthly income were not determinants of good BP control. In a study carried out in Ghana, Ogedengbe *et. al.* showed that the provision of health insurance coverage alone might not achieve the desirable outcome except if there was an additional intervention^30^. Summarily, this study has shown that the use of mHealth led to an improvement in the control of hypertension and this will lead to better treatment outcomes.

### Limitations of this study:

The differences in monthly income, and level of education may have contributed to the observed better BP control among the intervention group.

On account of limited financial resources, we only relied on self-reported history of cardiovascular complications or suspected secondary hypertension.

## Conclusions

The mHealth intervention resulted in a significant SBP reduction rate and improvement in the BP control rate in the 6th month. However, improvement in adherence level was only obvious in the 3rd month and was not sustained in the 6th month. The addition of mobile health intervention may be extended for use in the national hypertension control plan. Female gender, formal education and being in the intervention arm were predictors of blood pressure control.

## Figures and Tables

**Table 1:** Socio-demographics characteristics of the study population

Variables	Control n (%) (N = 109)	Intervention n (%) (N = 116)	p-value
**Age (Years)**			
< 45	7 (6.4)	2 (1.7)	0.300
45–54	22 (20.2)	25 (21.6)	
55–64	40 (36.7)	49 (42.2)	
≥ 65	40 (36.7)	40 (34.5)	
Mean ± SD (years)	60.2 ± 13.5	62.6 ± 10.8	
**Sex**			
Males	41 (37.6)	41 (35.3)	0.724
Females	68 (62.4)	75 (64.7)	
**NHIS Status**			
Enrollees	54 (49.5)	63 (54.3)	0.512
Non-Enrollees	55 (50.5)	53 (45.7)	
**Monthly Income (dollars)**			
≤ 100	90 (82.6)	77 (66.9)	0.002*
101–200	14 (12.8)	18 (15.5)	
201–300	2 (1.8)	4 (3.4)	
301–400	3 (2.8)	18 (15.5)	
> 400	0 (0.0)	3 (2.6)	
**Education status**			
No formal education	12 (11.0)	8 (6.9)	< 0.001*
Primary	24 (22.0)	15 (12.9)	
Secondary	52 (47.7)	30 (25.9)	
Tertiary	21 (19.3)	63 (54.3)	
**Occupation**			
Unemployed	13 (11.9)	12 (10.3)	0.001*
Self-employed	51 (46.8)	34 (29.3)	
Government employee	8 (7.3)	32 (27.6)	
Retired	27 (24.8)	28 (24.1)	
Non-government employee	10 (9.2)	10 (8.7)	
**Marital Status**			
Single	13 (11.9)	20 (17.2)	0.135
Married	70 (64.2)	77 (66.4)	
Separated	2 (1.8)	6 (5.2)	
Divorced	7 (6.4)	3 (2.6)	
Widowed	17 (15.6)	10 (8.6)	

Keys: NHIS, National Health Insurance Scheme

* Statistically significant.

**Table 2:** Comparison of pre-intervention and post-intervention of clinical parameters

Variable	Baseline Mean ± SD			At 6 months Mean ± SD		
	Control N = 109	Intervention N = 116	p-value	Control N = 109	Intervention N = 115	p-value
Mean pulse rate (bpm)	82.9 ± 17.0	84.4 ± 16.9	0.508	81.4 ± 13.7	79.7 ± 12.0	0.324
Mean SBP (mmHg)	160.1 ± 22.7	157.3 ± 19.6	0.322	156.2 ± 23.0	138.6 ± 16.5	< 0.001*
Mean DBP (mmHg)	91.8 ± 14.7	93.1 ± 12.4	0.473	89.3 ± 14.4	85.8 ± 9.4	0.032*
Weight (kg)	75.4 ± 16.3	77.4 ± 16.7	0.365	75.6 ± 16.1	78.6 ± 19.4	0.211
BMI (Kg/m^2^)	30.3 ± 17.2	28.8 ± 6.7	0.384	29.8 ± 16.2	29.0 ± 8.2	0.639
Waist Circumference (cm)	96.4 ± 13.7	95.7 ± 14.6	0.712	94.9 ± 13.9	93.7 ± 13.1	0.507
Hip Circumference (cm)	105.4 ± 12.0	105.1 ± 14.1	0.864	102.9 ± 11.6	102.7 ± 12.7	0.902
Waist-hip ratio	0.92 ± 0.09	0.91 ± 0.10	0.432	0.92 ± 0.10	0.91 ± 0.10	0.455

Keys: SBP, standard deviation; SBP, systolic blood pressure; DBP, diastolic blood pressure; BMI, body mass index

* statistically significant.

**Table 3:** Comparison of blood pressure control among study population at baseline, 3rd month and 6th month

Variables	Control n (%) (N = 109)	Intervention n (%) (N = 116)	p-value
**Baseline**			
Good BP Control	19 (17.4)	13 (11.2)	0.182
Poor BP Control	90 (82.6)	103 (88.8)	
**3rd Month**			
Good BP Control	26 (23.9)	41 (35.7)	0.054
Poor BP Control	83 (76.1)	75 (64.3)	
**6th Month**			
Good BP Control	27 (24.8)	51(44.3)**	0.002*
Poor BP Control	82 (75.2)	64 (55.7)	

Keys: BP, Blood pressure

* Statistically significant

** one patient died before his 6th month follow-up

**Table 4:** Comparison of medication adherence level of the study population at baseline, 3rd month and 6th month

Variables	Control n (%) (N = 109)	Intervention n (%) (N = 116)	p-value
**Baseline**			
Adherent	58 (53.2)	58 (50.0)	0.566
Non-Adherent	51 (46.8)	58 (50.0)	
**3rd Month**			
Adherent	63 (57.8)	78 (67.3)	0.029*
Non-Adherent	46 (42.2)	38 (32.8)	
**6th Month**			
Adherent	64 (58.7)	73 (62.9)	0.101
Non-Adherent	45 (41.3)	43 (37.1)	

Key:

* Statistically significant

**Table 5:** Relationship between good blood pressure control and socio-demographic variables at 6th month

	BP Control at 6 months		
Variables	Controlled n (%)	Uncontrolled n (%)	p-value
**Age (Years)**			
< 45	2 (22.2)	7 (77.8)	0.224
45–54	15 (31.9)	32 (68.1)	
55–64	38 (42.7)	51 (57.3)	
≥ 65	23 (29.1)	56 (70.9)	
**Sex**			
Males	20 (24.7)	61 (75.3)	0.017*
Females	58 (40.6)	85 (59.4)	
**National Health Insurance Scheme**			
Enrollees	42 (35.9)	75 (64.1)	0.724
Non-Enrollees	36 (33.6)	71 (66.4)	
**Monthly Income ($)**			
≤ 100	53 (32.7)	109 (67.3)	0.141
101–200	9 (28.1)	23 (71.9)	
201–300	2 (33.3)	4 (66.7)	
301–400	12 (57.1)	9 (42.9)	
> 400	2 (66.7)	1 (33.3)	
**Education status**			
No formal education	1 (5.0)	19 (95.0)	0.010*
Primary	10 (26.3)	28 (73.7)	
Secondary	32 (39.0)	50 (61.0)	
Tertiary	35 (41.7)	49 (58.3)	
**Occupation**			
Unemployed	8 (32.0)	17 (68.0)	0.118
Self-employed	26 (30.6)	59 (69.4)	
Government employee	18 (45.0)	22 (55.0)	
Retired	15 (27.8)	39 (72.2)	
Non-government employee	11 (55.0)	9 (45.0)	
**Marital Status**			
Single	17 (51.5)	16 (48.5)	0.052
Married	50 (34.2)	96 (65.8)	
Separated	0 (0.0)	8 (100.0)	
Divorced	4 (40.0)	6 (60.0)	
Widowed	7 (25.9)	20 (74.1)	
**Cohort**			
Control	27 (24.8)	82 (75.2)	0.002*
Intervention	51 (44.3)	64 (55.7)	

Key:

* Statistically significant

**Table 6:** Predictors of blood pressure control at 6th month

Variable	AOR	95% Confidence Interval		p-value
		Lower	Upper	
**Sex**				
Males (ref)	1.00			
Females	2.52	1.33	4.79	0.005*
**Education status**				
No formal education (ref)	1.00			
Primary	8.69	1.00	75.58	0.050*
Secondary	16.61	2.06	133.91	0.008*
Tertiary	15.07	1.86	121.76	0.011*
**Cohort**				
Control (ref)	1.00			
Intervention	2.33	1.23	4.42	0.009*

Key: AOR, adjusted odd ratio

## Data Availability

All data will be made available upon reasonable request from the corresponding author, Dr Tijani Idris Ahmad., Department of Family Medicine, Ambrose Alli University, Ekpoma, Nigeria. E-mail: tijanioseni@aauekpoma.edu.ng.

## Abbreviations

BP: Blood Pressure
SBP: Systolic Blood Pressure
mHealth: Mobile Health
SSA: Sub-Saharan Africa
OPD: Outpatient Department
MH: Mamprobi Hospital
AP: Adabraka Polyclinic
FTH: Federal Teaching Hospital
BMI: Body Mass Index
WHR: Waist-to-Hip Ratio
JNC-7: Joint National Committee on Prevention, Detection, Evaluation, and Treatment of High Blood Pressure- Seventh
SD: Standard Deviation
DBP: Diastolic Blood Pressure
AOR: Adjusted Odd Ratio
